# The hybrid models, containing hydrolytic and electron-driven processes, in theoretical study of oxaliplatin biotransformation

**DOI:** 10.1007/s00894-020-04549-4

**Published:** 2020-09-26

**Authors:** Janina Kuduk-Jaworska, Jerzy J. Jański, Szczepan Roszak

**Affiliations:** 1 Faculty of Chemistry, Wrocław University, F. Joliot-Curie 14, 50-370 Wrocław, Poland; 2 Department of Physical and Quantum Chemistry, Faculty of Chemistry, Wrocław University of Science and Technology, Wyb. Wyspiańskiego 27, 50-370 Wrocław, Poland

**Keywords:** Oxaliplatin transformation, Electron-driven reactions, DFT calculations, Hybrid mechanism, Nonclassical Pt-water interactions

## Abstract

The results of theoretical simulations of reaction paths for oxaliplatin from pro-drug into its active form responsible for cytostatic effect are presented. The studies based on the quantum-chemical density functional theory approach were performed considering environmental influence resulting from the aquation or electron donation. The hybrid mechanisms: hydrolytic mixed with electron driven were found to be the energetically favourable.

**Electronic supplementary material:**

The online version of this article (10.1007/s00894-020-04549-4) contains supplementary material, which is available to authorized users.

## Introduction

**Fig. 1:**
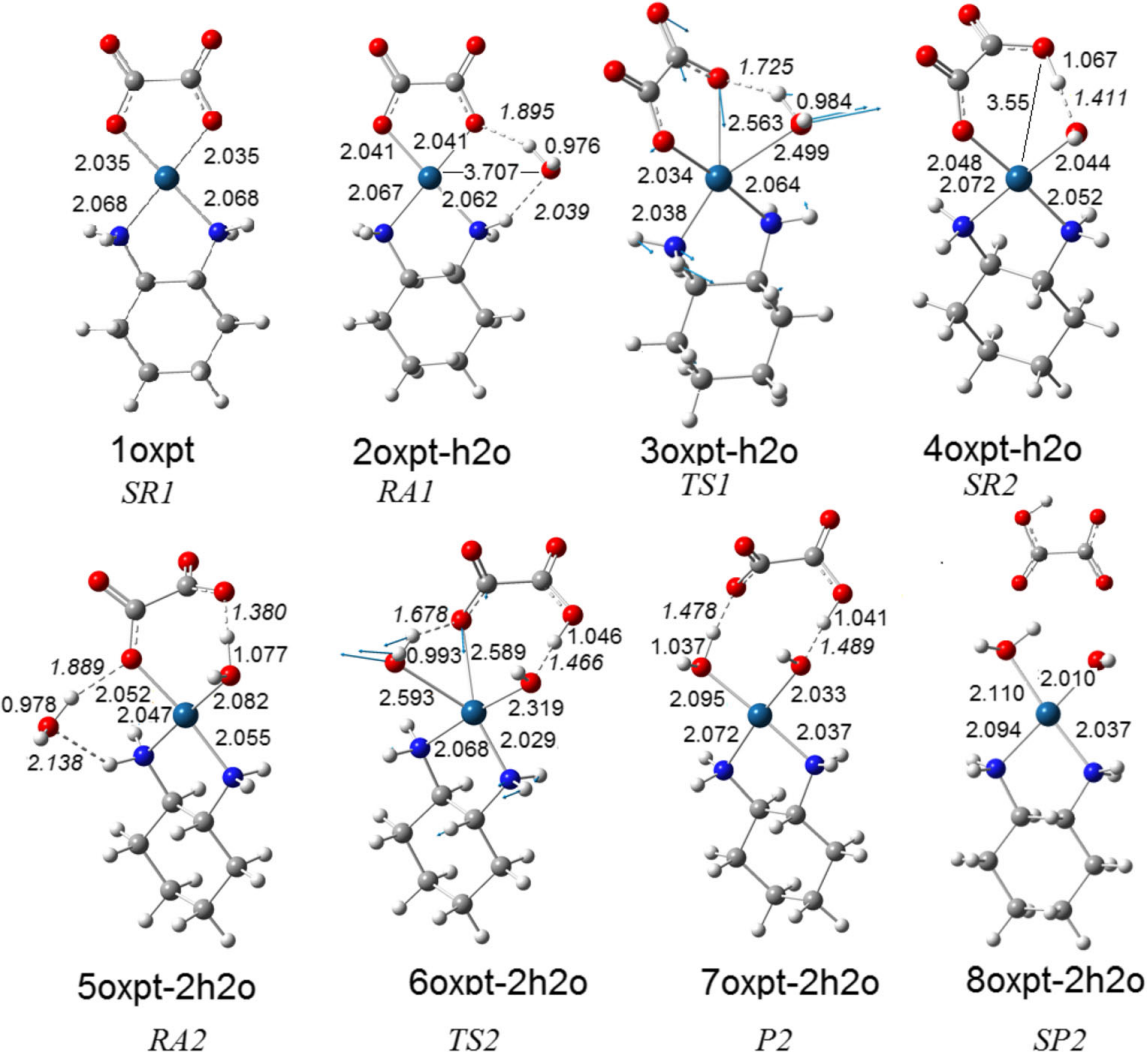
Optimized structures of the species involved in the hydrolysis of oxaliplatin. The included distances are given in angstroms

The costly and time-consuming studies, including chemical, physical, biological and pharmacological procedures, are necessary to establish if the evaluated compound can be introduced into the market as an approved drug. Statistically, only one in 5000 to 10,000 compounds directed to in vitro tests becomes officially recognized drug [1]. The search for anticancer drugs proved more efficient in metal compounds, but the results are not satisfying either. So far, over 3000 platinum coordination compounds have been evaluated in vitro, out of which three compounds were selected and registered worldwide for use in the standard treatment of cancer [2]. Oxaliplatin (IUPAC Name: (1R,2R)-cyclohexane-1,2-diamine;oxalate;platinum(2+)) constitutes a platinum-based drug of the latest generation. This medicine, as Eloxatin™ for injection in combination with infusional 5-fluorouracil and leucovorin, was approved by FDA in 2002 for the treatment of patients with metastatic carcinoma of the colon or rectum. It was introduced by the USA in 2002, but earlier was registered for the treatment of advanced colorectal cancer (ACRC) in France (1996) and by European Union (1999) [3, 4]. Oxaliplatin (Fig. 1), similarly as two earlier available drugs, cisplatin and carboplatin, belongs to the family of neutral platinum(II) complexes, characterized by the square motive at the Pt center. The important advantage of oxaliplatin is the lack of tumour cross-resistance with cisplatin and carboplatin, which reinforced the belief in Pt complexes as the proper area of exploration for the new generation of anticancer drugs. However, oxaliplatin is not free from adverse effects. Interestingly, all three Pt-based compounds, though demonstrating serious shortcomings and limited activity against many forms of human cancer, are still in the range of the most effective agents in the cancer therapy. It means that the search for new, more efficient chemicals continues to be a real challenge.

The aim of modern approaches is to reduce the cost and time required for the production of drugs capable of selectively killing cancer cells without triggering unwanted side effects. Thus, there is a need to improve a drug development cycle which starts from a meticulously designed chemical compound and leads to a form which will turn out to be a reliable candidate for the FDA approval. Undoubtedly, the detailed knowledge of the biological and biochemical fate of the drug in a living organism will be helpful in the quest for anticancer “magic bullet”. The therapeutic action of oxaliplatin is still not sufficiently understood. Generally, it is accepted that the mechanism of oxaliplatin biotransformation emulates the hydrolytic processes known for cisplatin and carboplatin [5, 6]. Thus, the aqua-products are supposed to constitute an active form of drug. It is noteworthy that arguments for such a hypothesis were provided by Videhult [7] who synthesized the pure diaqua oxaliplatin especially to evaluate its activity. In fact, the prepared compound was more cytotoxic than intact oxaliplatin, proving that antineoplastic action of drug might be mediated through diaqua-complex, identical with the final product of oxaliplatin biotransformation. It was also postulated that diaqua oxaliplatin, similarly as the highly reactive Pt-aqua-complexes derived from cisplatin and carboplatin, is capable of exchanging its water ligands in reactions with N-donor sites on DNA and forming the same type adducts [5, 8]. However, despite the similarity in biochemical processes in the transformation of the three Pt-based pro-drugs, the profiles of their biological and pharmacological activity differ significantly [8, 9]. This discrepancy suggests that not only the metabolic cycle and the DNA crosslink formation, but also other, more subtle interactions of active species with different molecular targets, can influence heavily the biological activity [5].

In the present work, we are concentrated on the processes preceding the DNA platination and try to characterize the unique metabolic course of oxaliplatin, believing that the understanding of transformation mechanism is crucial to predict the character of interactions between active drug and biological targets. For Pt-containing cytostatics, the formation of the activated form begins with the impact of nucleophiles (e.g. water, e-donor) on the central ion. The further course of metabolic reactions of drug depends on the character of the Pt(II) center, which is moulded by coordinated ligands. Consequently, the sequence of reactions, including breaking and creating of bonds, is controlled by stereo-chemical and electronic properties of ligands. Their multi-factorial influence manifests itself in the determination of the specificity in biotransformation of this pro-drug and a high selectivity in the reaction of the real drug with a molecular target. Since oxaliplatin belongs to the same family of neutral Pt(II) complexes as two other platinum(II) drugs, it may be considered as a hypothetic course of oxaliplatin transformation, but cannot be a sufficient premise to expect close similarities in mechanistic courses of that pro-drug activation. The similarities and differences calculated for individual drugs can be used in comparative studies on the non-enzymatic metabolism of that group of compounds.

This work, which is the continuation of our earlier studies on cisplatin and carboplatin biotransformation [10, 11], aimed at enhancing our knowledge on processes underlying the medicinal action of oxaliplatin. The idea and the methodology applied previously were also used to study the oxaliplatin metabolism. The investigations were performed implementing the quantum-chemical simulations at the density functional theory (DFT) level methods as described earlier [10–18]. The satisfied conformity between our calculated and the earlier measured [19] structure of the oxaliplatin molecule indicates that the chosen in silico methodology is reasonable. Up to date, the theoretical approach responding to mechanistic questions of the oxaliplatin activation was implemented adopting only the hydrolytic mode of transformation [12, 13, 18]. The present work supports earlier studies by describing the whole cycle of the non-enzymatic oxaliplatin transformation. Two mechanistic models: (1) based on paradigm of aquation, corroborated yet for other Pt-based drugs, and (2) based on the electron-transfer are considered. The simulation of metabolic processes, triggered by the impact of electron on oxaliplatin, has been performed here for the first time, revealing new mechanistic pathways of pro-drug transformation, different from the hydrolytic pathways. More specifically, it was confirmed that oxaliplatin can be metabolized by hydrolysis, which proceeds via the associative mechanism, as well as by the sequence of reactions driven by electron-transfer (ET) processes. Though these two mechanistic courses are competitive and the ET route was found as more favourable, we do not rule out a “pure hydrolytic” pathway. Moreover, we advanced an argument to postulate that the ET events coupled with hydrolytic reactions are the most favourable, leading to the hybrid mode of transformation. The theoretical studies allowed to reject of popular belief that (di)aqua-platinum(II) complexes are the only species ready to interact with DNA. Concluding, we can verify theoretical assumptions and explain which transformation cycles show to be the most favourable in the case of oxaliplatin metabolic degradation.

## Theoretical methods and computational details

The quantum-chemical calculations were performed within the density functional theory [20] with B3LYP functional [21, 22]. The applied functional has proven its reasonable performance for other systems containing platinum [10–18]. The atomic basis sets (1) 6–31++G(2df, 2pd) for light atoms [23, 24] and the quasi relativistic effective core potential and corresponding basis set proposed by the Stuttgart group were used for platinum atom [25], for which 18 electrons were kept in the valence space and (2) def2-TZVP [26] were applied. The minimum-energy structures and transition states in the respective cases were confirmed through frequency calculations. No symmetry constrains were assumed at the optimization stage of calculations. The calculations were performed for the gas phase and water solvent environment. Solvent effects were included using the COSMO [27]. Calculations of thermodynamic properties were carried out by applying the ideal gas, rigid rotator, and harmonic oscillator approximations [28]. All of the computations were carried out using Gaussian 16 code [29]. Molecular graphics have been generated using the GaussView06 software [30].

## Results and discussion

Like most medicines, platinum-based drugs are administered to patients in the form which does not allow a therapeutically effective interaction with a target bio-molecule. Thus, the drug in this form, known as pro-drug, requires transformation into active metabolite prior to the reaction with cellular target. The transformation of pro-drugs belonging to Pt(II)-complexes relies mainly on the substitution of coordinated labile ligands with nucleophiles present in both extracellular and intracellular milieu. Consequently, many processes can be considered as potential transformation pathways: hydrolysis, substitution with chloride, phosphates, carbonates and sulphur-containing compounds. However, the vast majority of studies on platinum drug metabolism are concentrated only on the impact of water, while the influence of other potential bio-reagents remains to be determined. In general, the hydrolysis of platinum pro-drugs is recognized as the only way of bio-activation and the aquated complexes as the only metabolites causing irreparable DNA damage [31]. This fact was also used to explain the oxaliplatin bio-activation. The hydrolytic mechanism suggested herein was consistent with the data obtained from many experimental [7, 32, 33] and a few theoretical studies [12, 13] which showed that the di-aqua-oxaliplatin complex, the final product of nucleophilic substitution, is the active species ready to interact with DNA and develop the cytotoxic effect. However, these results do not rule out a scenario where oxaliplatin can be simultaneously metabolized along other mechanistic pathways, particularly in the fashion documented for related platinum drugs (cisplatin and carboplatin) [10, 11]. It seems therefore reasonable to assume that the bio-activation of oxaliplatin is a multi-channel process, where low energy electrons (LEE) may play an important, aqua-assisting, role. To create the cycle of oxaliplatin transformation for in silico procedures, several models characterizing the multi-step reactions are presented. The computational simulation verified the correctness of postulated reaction courses.

### Hydrolytic transformation of oxaliplatin

**Scheme 1:**
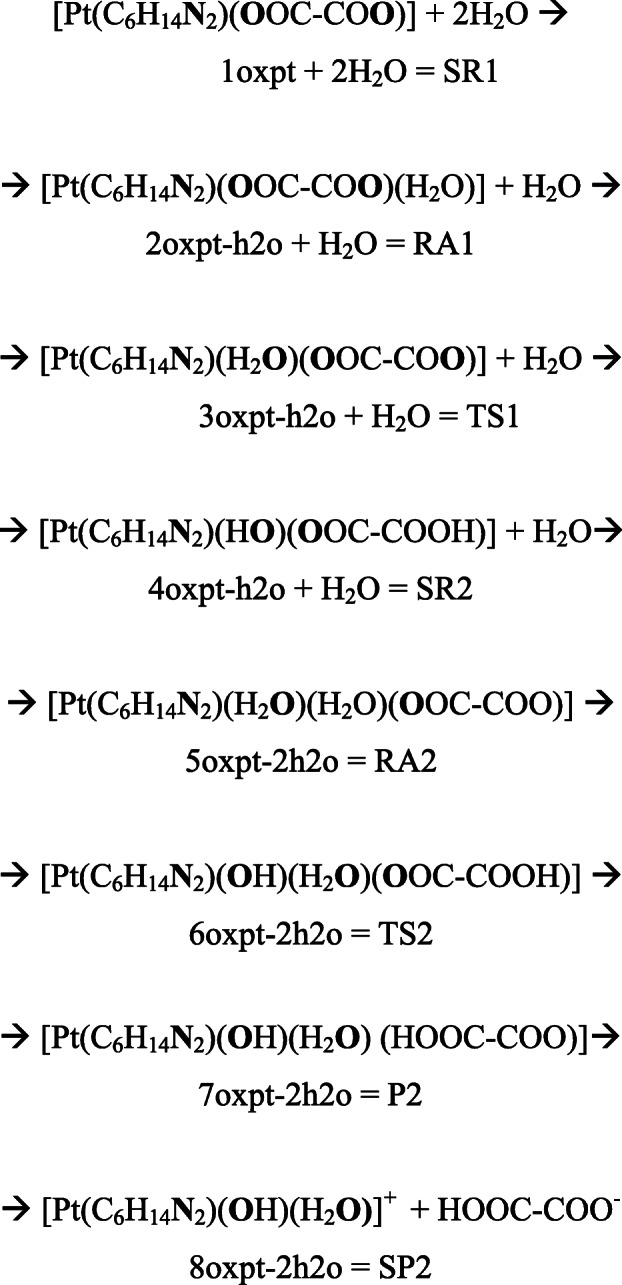
Reaction paths of two stage oxaliplatin hydrolysis (donor atoms in bold)

In order to understand the mode of oxaliplatin bio-activation, we followed the predicted course of hydrolytic transformation and performed quantum chemical calculations based on the super-molecular approach. The substrates for hydrolytic substitution consist of oxaliplatin and two water molecules treated as separated reactants. For the sake of methodological consistency, we carried out a theoretical analysis of oxaliplatin hydrolysis, comparing the results with the ones reported earlier for the same chemical system and applying similar procedures [12]. The sequence of reactions in hydrolytic course, estimated on the basis of computational simulation, confronted with structures of reactants (Fig. 1) is shown in Scheme 1.

#### Structural analysis of reaction species

The transformation (Scheme 1) begins with water impact on an oxaliplatin molecule and continues in two consecutive stages. Both stages are characterized by a sequence of stationary states, related to the mechanism of a subsequent exchange of two anionic groups, running via the five-coordinate transition state. Generally, as it is demonstrated in Table 1S, data for structural parameters obtained by a different computational treatment does not differ significantly. For clarity, we discuss only geometrical parameters of stable states presented on Fig. 1, based on calculations which adopted the B3LYP/def2TZVP/SCRF=CPCM model.

**Fig. 2:**
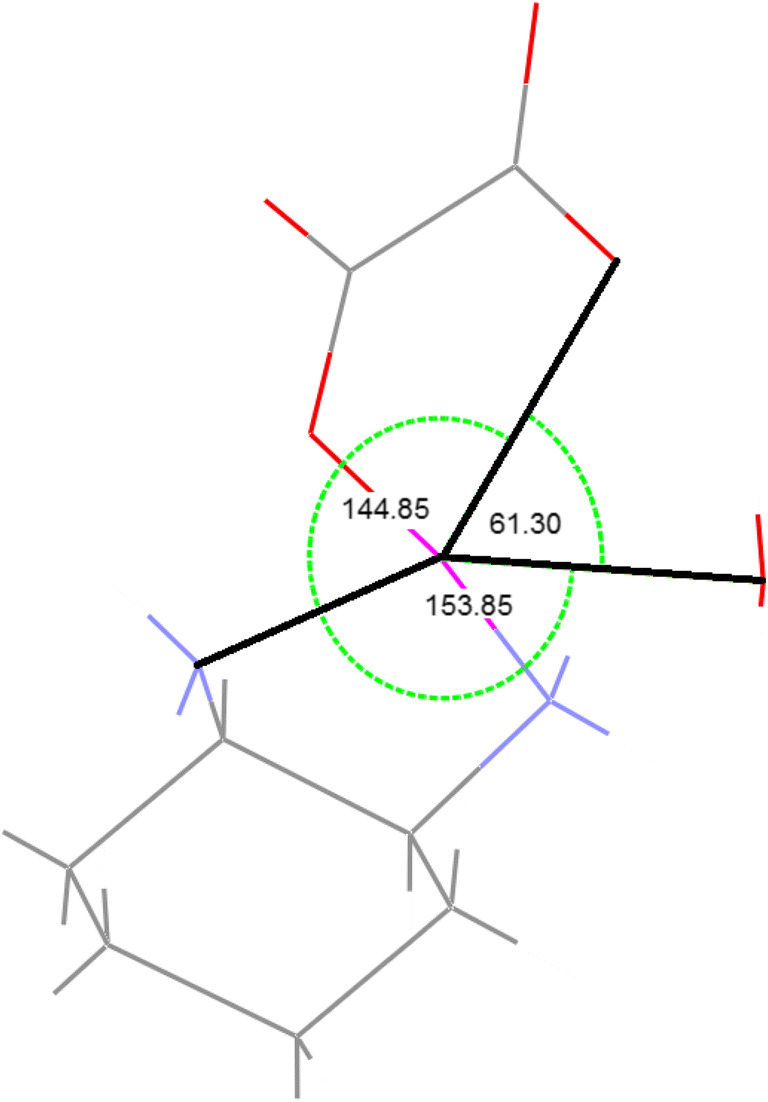
The structure of the first transition state (Fig. 1; TS1) for the two-stage hydrolysis of oxaliplatin. The sum of concentric angles at the Pt-center amounts to 360°

The interaction of oxaliplatin and H_2_O molecule leads first to the formation of super-molecular adduct (2oxpt-h2o), which is stabilized by two hydrogen bonds: one between the entering water molecule and H of NH_2_ group (2.037 Ǻ) and the other between the same water molecule and O of oxalate anion (1.893 Ǻ); no binding occurs between H_2_O and Pt(II) center (3.705 Ǻ). The further transformation of 2oxpt-h2o into 3oxpt-h2o entails the formation of a distorted trigonal-bipyramid (TBP), a geometry characteristic for a transition state. During this conversion, the distance between Pt-center and O atom of incoming H_2_O decreases from 3.705 to 2.498 Ǻ, whereas the breaking bond of Pt–O (oxalate) increases from 2.040 to 2.561 Ǻ. With this geometry, the entering water molecule and the leaving COO^−^ group, together with NH_2_ of non-labile ligand, occupy equatorial positions in the plane, including the metal ion in the center. The axial positions are occupied by other NH_2_ and by resting carboxylate group belonging to dianionic chelate. The coplanar position of N, Pt, O (of water), and O (of oxalate) in the TS1 structure was confirmed by the sum of three concentric angles, whose total is close to 360° (Fig. 2). The nature of the transition state of 3cbp-h2o was also verified by the frequency analysis. A single imaginary frequency of 193i cm^−1^ was assigned as an anti-symmetrical stretching vibration of Pt–O (oxalate) bond breaking, occurring simultaneously with the bond formation of Pt–O (water). The spontaneous conversion of 3oxpt-h2o provides a neutral mono-hydroxo complex 4oxpt-h2o, marked by the proton transfer from the water molecule to carboxylate anion. The product of the first-stage hydrolysis is at the same time a substrate which triggers the second stage of oxaliplatin transformation. Its further interaction with water molecule leads to the formation of 5oxpt-2h2o adduct, which is stabilized by three hydrogen bonds, one created by H_2_O bound with Pt(II): (H_2_O)**H**…**O**(COO^−^) (bond length 1.384 Ǻ), and the other two by entering H_2_O: (NH_2_)**H**…**O**(H2O)**H**…**O**(COO^−^) (bond distances 2.138 and 1.889 Ǻ), respectively). The added water molecule is situated outside the first coordination sphere and is separated from the metal center by 3.613 Ǻ. Then, the 5oxpt-2h2o turns into a transition state TS2 (equal to 6oxpt-2h2o) of a five-coordinate structure, in which the entering H_2_O and the leaving COOH occupy equatorial positions in the plane of TBP geometry. The distance between Pt and O atom of the incoming H_2_O declines from 3.613 to 2.593 Ǻ, whereas the breaking Pt–O bond increases from 2.051 to 2.587 Ǻ. Similarly to TS1, TS2 is characterized by the sum of nearly 360° for the concentric angles in the co-planar position formed at Pt-center.

The positive verification of the transition state character of 6oxpt-2h2o was provided by the analysis of imaginary frequency level of *ν*_imag_ = 195i cm^−1^, which can be attributed to the anti-symmetrical stretching vibration of Pt–O(oxalate) bond breaking, occurring simultaneously with the Pt–O(water) bond formation. The conversion of 6oxpt-2h2o can lead to the final product of substitution (7oxpt-2h2o), and further to separated species (8cbp-2h2o).

#### Hydrolysis of oxaliplatin-energetic effects

**Fig. 3:**
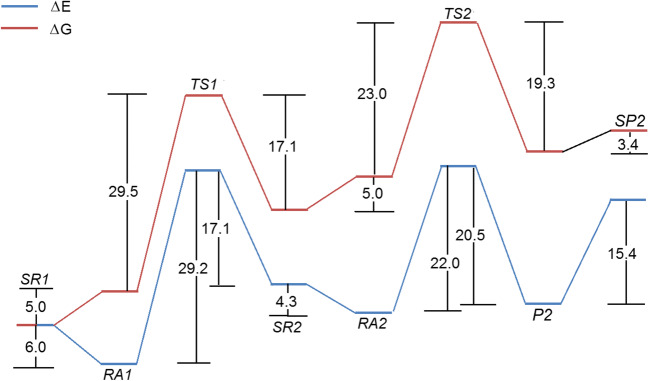
The reaction profile for the hydrolysis of oxaliplatin for structures given on Fig. 1. The energies (Δ*E*) and free energies (Δ*G*) are in kcal/mol

The energy effects associated with oxaliplatin biphasic hydration were computed following the reaction courses presented in Scheme 1. The energy parameters were calculated within the B3LYP/def2TZVP/SCRF=CPCM procedure, the ones that were used also for geometry. The results are shown on Fig. 3.

As observed on Fig. 3, the internal energy of the starting reaction state (SR1) is lowered after accepting water molecule and formation of the RA1 system that contains 2oxpt-h2o adduct and free water molecule. The transition from the RA1 to TS1 is associated with the energy consumption of 29.2 kcal/mol, but the activation barrier, defined by some authors [34], as the difference in the energy between the transition state and the separate reactants state, amounts here to 23.2 kcal/mol. In subsequent steps, the transition state TS1 undergoes the spontaneous transformation into SR2, a complex including the 4oxpt-h2o product and water molecule. The interaction of both components initiates the second stage of carboplatin hydrolysis. It begins with exoenergetic (− 4.3 kcal/mol) process SR2 ➔ RA2 leading to the adduct, the supermolecule 5oxpt-2h2o, which is transformed through TS2 into P2, the final product of the two-stage hydrolysis of oxaliplatin. The activation barrier of SR2 ➔ TS2 amounts to 22.0 kcal/mol and is significantly lower than the relevant effect (29.2 kcal/mol) in the first stage. Thus, it can safely be concluded that the first aquation is the rate-determining stage, which provides species capable of easily undergoing a further substitution. As a result, the main product is a fully hydrolyzed complex 7oxpt-2h2o, which is rather stable, as indicated by the evaluation of its further hypothetical transformation. Theoretically, two separated species, [Pt(C_6_H_14_**N**_2_)(**O**H)(H_2_**O**)]^+^ and HOOC-COO^−^ (8cbp-2h2o, Fig. 1), could be created after the endo-energetic (15.4 kcal/mol, Fig. 3) breakdown of two hydrogen bonds in the 7oxpt-2h2o supermolecule. The inclusion of entropy effects leads to the qualitatively same picture.

Concluding: although the supermolecule 7oxpt-2h2o might be recognized as an active form of the drug, we nevertheless cannot rule out the other candidates in the competition, namely: (i) the mono-hydroxo, mono-oxalato-oxaliplatin (4oxpt-h2o), (ii) the cation of mono-aqua-mono-hydroxo-oxaliplatin, and (iii) intact oxaliplatin. Moreover, the key features constitute not only the energetic relations between the transformed species but also the energy effects of their transport and interaction with the DNA, or other targeting bio-molecules. As long as these data are unknown, we cannot determine which of these Pt-complexes are the true active forms of oxaliplatin. Additionally, the reach environment of the reaction provides other, more exotic reaction paths which may not be ignored.

### Oxaliplatin transformation based on the electron-driven process

This investigation constitutes the continuation of our work on platinum-drug transformation [10, 11], which is based on the concept of influential role of electron transfer (ET) events in metabolic processes [35]. The ET-driven concept showed satisfactory conformity with both experimental and theoretical research on mechanistic aspects of cisplatin and its congeners activation [4, 10–12, 36]. The designed reactant originating from irradiated water solution is the pre-hydrated electron (*e*^−^_pre_), the component of “secondary products”, created in the physical stage of water radiolysis. From the starting point, these electrons are endowed with a large amount of kinetic energy with the effect that collisions with their potential acceptors do not result in the electron capture. However, after losing the energy via ionization and excitation events, the ultrashort-lived (~ 10^−15^ s) electrons become thermalized and retain their energy at the level of ~ 0–20 eV, characteristic for the low energy electrons (LEEs) [37]. The LEEs, contrary to higher energy electrons, are capable of triggering chemical selective e-burden reactions. In general, they are very reactive with water and some biological molecules, including DNA. They can also be captured by xenobiotic molecules, such as synthetic pesticides or natural polyphenolic compounds, which are known for their antioxidant properties [38]. Low-energy electrons can also exist in non-irradiated biological systems. Particularly, the mitochondrial cycle produces high-energy electrons that enter the “electrons-transport chain”, where a series of electron carrier molecules move the electrons to a lower energy level. This process enables the electrons to react effectively with acceptor molecules. Assuming that LEEs from irradiated and non-irradiated biological systems can react with e-acceptors under the same mode, it is obvious that anticipated transformation of oxaliplatin can be carried out independently of the source of non-hydrated electrons. Given that cisplatin and carboplatin, analysed in silico, are good electron acceptors, we believe that oxaliplatin is a proper subject to conclude a study on the influential role of excessive electron on metabolic pathways of FDA-approved platinum anticancer drugs. Although all three compounds belong to the same class of metal-containing drugs, they differ considerably in biological activity, constituting a scientifically attractive option to look at when exploring mechanistic and “structure-activity” relations.

**Fig. 4:**
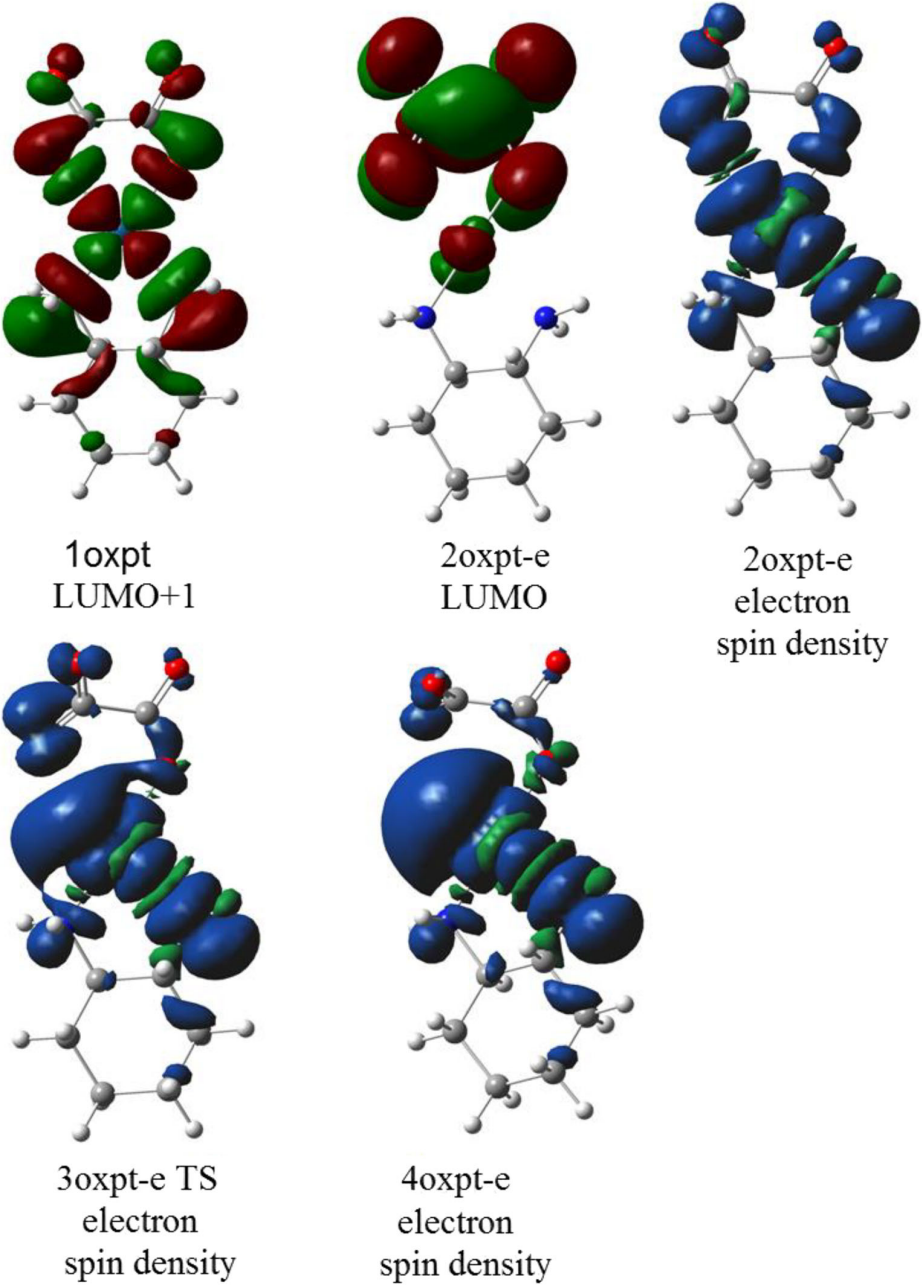
The unsymmetrical evolution of electron spin density (Fig. 7; top) resulting from electron attachment via the LUMO+1 orbital. The 2oxpt-e LUMO picture indicates the orbital not active in this reaction. The corresponding structures are found on Fig. 7

**Fig. 5:**
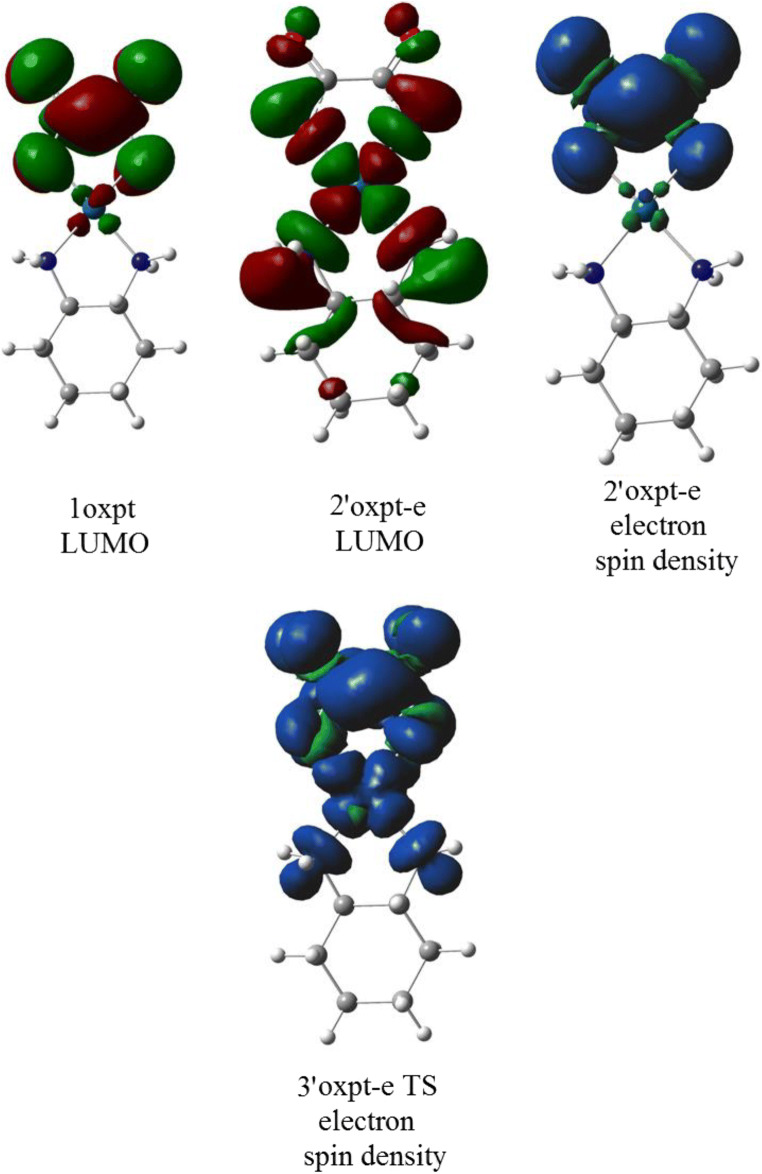
The symmetric evolution for the valence type of electron attachment to LUMO of oxaliplatin. The 2’oxpt-e LUMO indicates inactive in this path molecular orbital of an anion

**Fig. 6:**
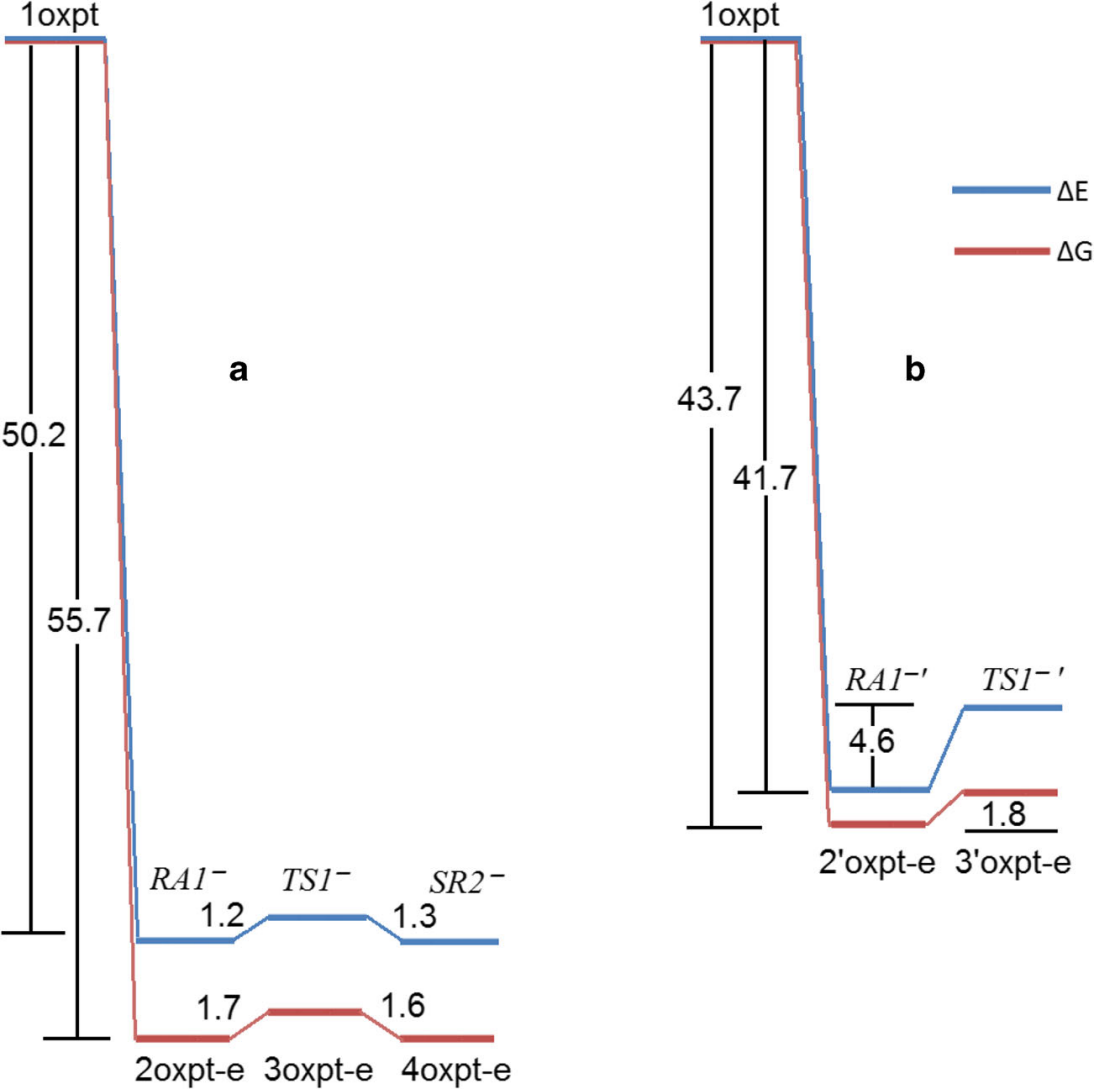
The reaction profiles for the electron attachments proceeding via LUMO+1 (**a**) and LUMO (**b**) orbitals of oxaliplatin. Energies (Δ*E*) and free energies (Δ*G*) in kcal/mol

**Fig. 7:**
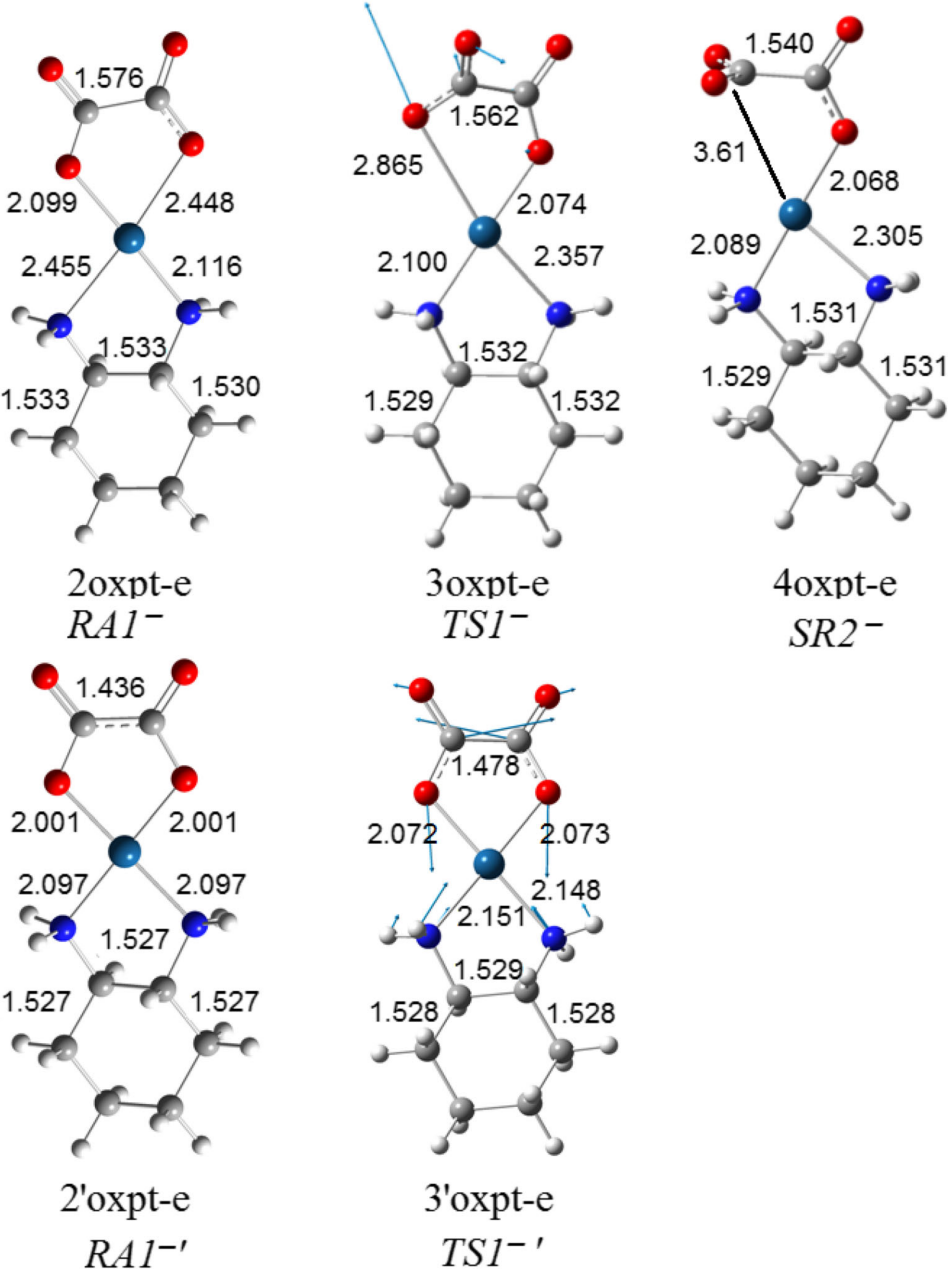
The structures of anions resulting from the electron attachment to oxaliplatin. The top row (RA1^−^–SR2^−^) corresponds to the unsymmetrical evolution. The bottom (RA1^−^′–TS1^−^′) characterizes the symmetrical path. Distances in angstroms

The orbital energies of two lowest unoccupied molecular orbitals of oxaliplatin are close in value (~ 5 kcal/mol difference); however, LUMO and LUMO+1 differ drastically in the localization and symmetry (Figs. 4 and 5). The electron attachment via LUMO+1 orbital leads to the energetically lowest anion with the spin electron density located in the Pt vicinity (Fig. 6). Although the total atomic charge on oxalate moiety increased by about one electron, the electron spin density on this fragment amounts to 0.11 electron only. The further rearrangement of anion structure, through the low lying transition state (Fig. 7), leads to the fully dissociated Pt–O bond and a very reactive complex. The oxalate constitutes the very negative fragment with the charge of 1.7 electron and electron spin density delocalized on the Pt part. The electron spin density and charge distributions make an anion vulnerable for the further dissociation reaction. The attachment of second electron is little probable, more important that leads to the dissociation of Pt-N bond instead Pt-O one.

The electron attachment via the lowest unoccupied orbital of oxaliplatin leads to the valence bound electron and the resulting anion preserves symmetry of the parent molecule. The electron spin density (0.997 electron) is entirely located on the oxalate moiety. The total atomic charge is also localized on this part. The transition state preserves the symmetry and the dissociation path involves both Pt–O bonds. However, the activation energy for this path is quite high and the reaction channel is not probable.

#### The hybrid hydrolytic and electron-driven processes

**Fig. 8:**
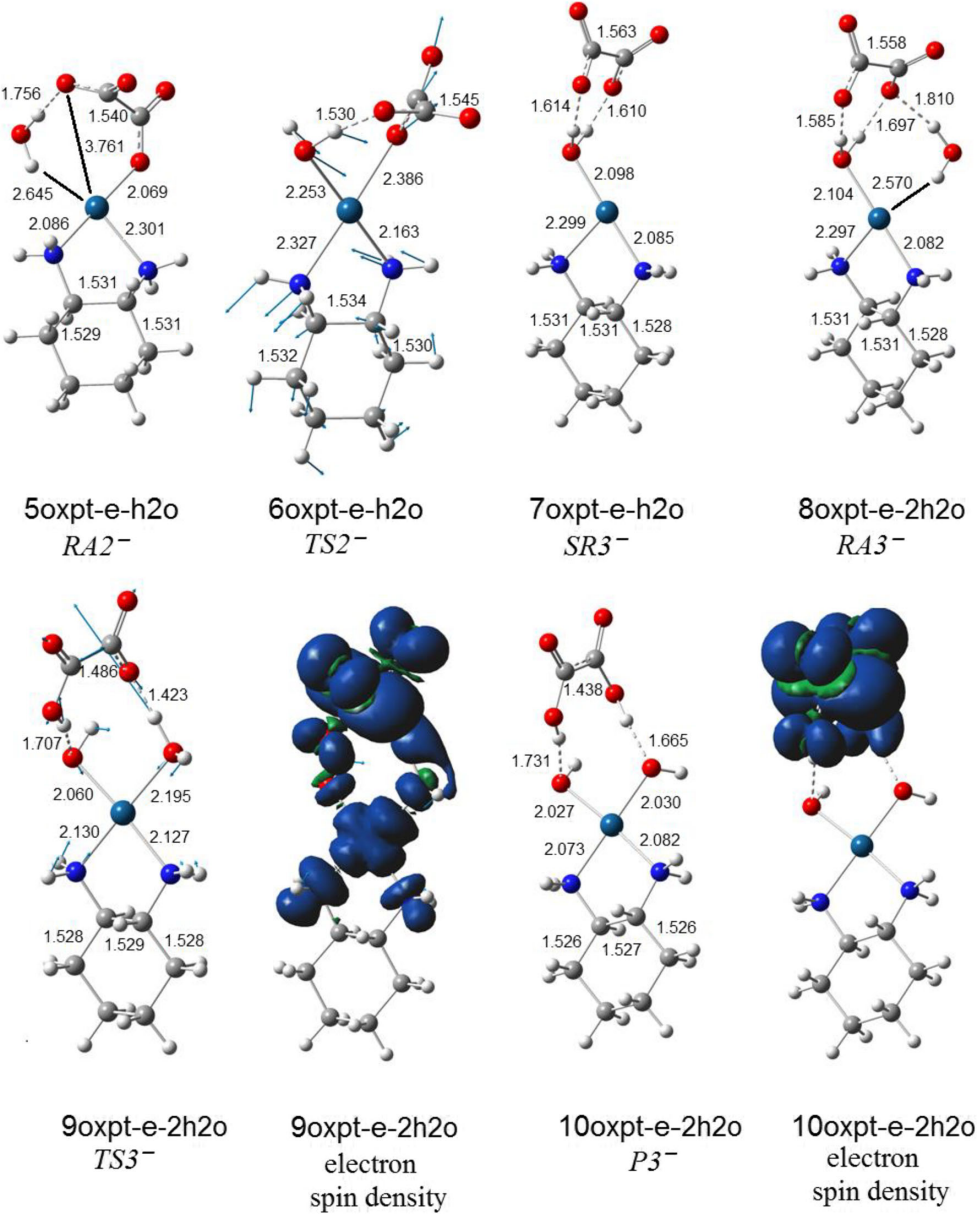
The hydrolytic path for the reaction of two consecutive water molecules with the most stable oxaliplatin anion (4oxpt-e on Fig. 7)

**Fig. 9:**
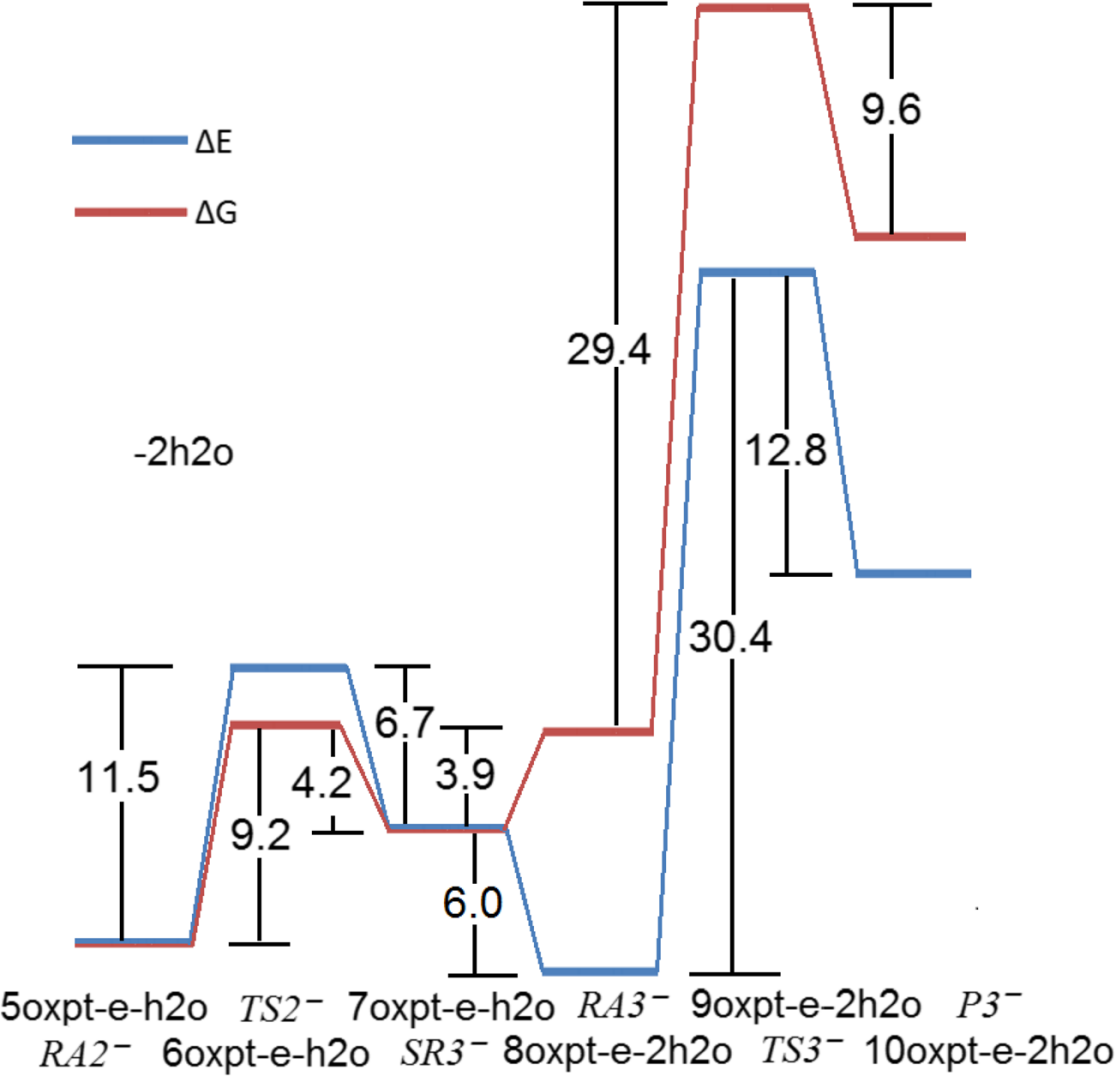
The energy (Δ*E*) and free energy (Δ*G*) profiles for the hydration of the most stable oxaliplatin anion (Fig. 8). Energies in kcal/mol

**Fig. 10:**
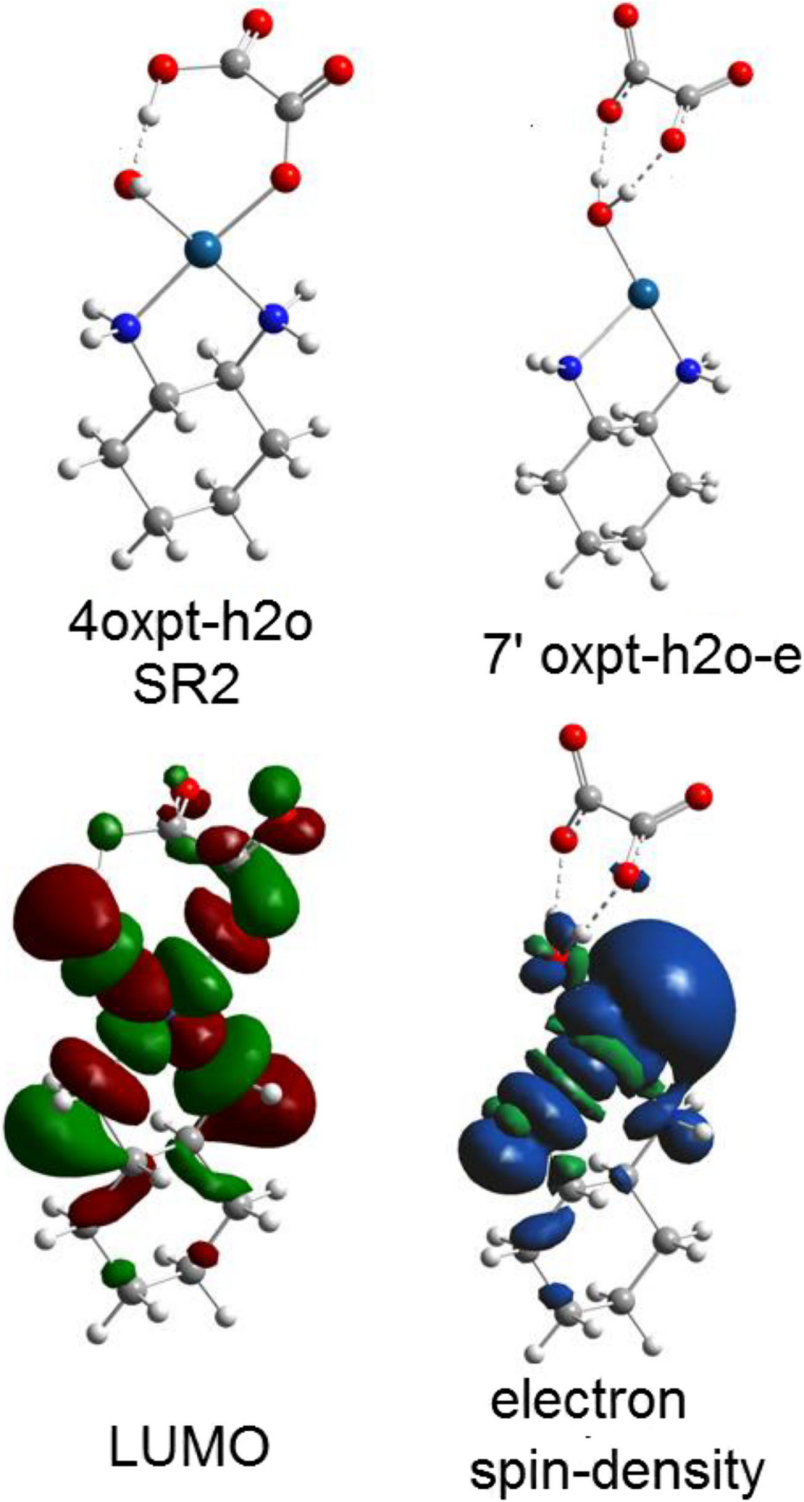
The hydrated oxaliplatin complex (Fig. 1) and resulting anion from the electron attachment. The parent structure LUMO and its corresponding electron spin density of an anion

The electron attachment to oxaliplatin resulting from the dissociation of the single Pt–O leads to the reactive anion which bounds the water molecule (Fig. 8). The resulting complex undergoes the transformation through the low-energy transition state to the intermediate product (Fig. 9). Interestingly, the electron attachment to the hydrated oxaliplatin leads to the same complex (Fig. 10) with the electron spin density localized on the Pt fragment. The further reaction with the second water molecule forms the 8oxpt-e-2h2o complex which undergoes the transformation into the well-separated anion-molecule complex (Fig. 8). The above process is characterized by the unpaired electron shift with electron localized on the oxalate fragment. However the observed pathway for the second water is no longer energetically favourable and is not competitive to the second part of the neutral hydrolytic transformation. The possible electron loss from 8oxpt-e-2h2o (Fig. 8) leads directly to the 7oxpt-2h2o neutral species (Fig. 1).

## Conclusions

In the present work, processes preceding the DNA platination are studied. The corresponding transformation mechanism is important to predict the character of interactions between active drug and biological targets. For Pt-containing cytostatics, the formation of the activation form begins with an impact of environmental components (such as water or active electrons) on the active chemicals (oxaliplatin in the present case). The further metabolic reactions of drug depends from the Pt(II) center character influenced by coordinated ligands.

The biotransformation of oxaliplatin was considered taking into account hydrolysis and processes stimulated by the electron transfer from the environment. The hydrolysis of cisplatin is based on two steps of the consecutive Pt–O dissociation (Fig. 3). The concerted single-step hydrolysis was not found. The electron attachment leads to the single Pt–O bond dissociation with a negligible activation energy. More important, the attachment of water molecule destroys the second Pt–O bond with the transition state energy reduced by half. The inclusion of entropy effects does not change the qualitative picture of energetic profiles for studied reactions.

## Electronic supplementary material

ESM 1 (DOCX 22 kb)
