# Supplementary material for: The hybrid models, containing hydrolytic and electron-driven processes, in theoretical study of oxaliplatin biotransformation
Source: J Mol Model. 2020 Sep 26;26(10):286. doi: 10.1007/s00894-020-04549-4 (PMC7519906; doi:10.1007/s00894-020-04549-4)
Supplement: Supplementary file 1 — (DOCX 22 kb) [file 894_2020_4549_MOESM1_ESM.docx]

**The hybrid models, containing hydrolytic and electron-driven processes, in theoretical study of oxaliplatin biotransformation**

**Janina Kuduk-Jaworska^a^, Jerzy J. Jański^a^, Szczepan Roszak^b*^**

^a^Faculty of Chemistry, Wrocław University, F. Joliot-Curie 14, 50-370 Wrocław, Poland

^b^Department of Physical and Quantum Chemistry, Faculty of Chemistry, Wrocław University of Science and Technology, Wyb. Wyspiańskiego 27, 50-370 Wrocław, Poland

Corresponding author, Szczepan Roszak, e-mail: [szczepan.roszak@pwr.edu.pl](mailto:szczepan.roszak@pwr.edu.pl)

Supplementary Data

**Table1S**

**Interatomic distances [Ǻ] in Pt- species formed in hydrolysis of oxaliplatin.**

| Transformed species of oxaliplatin | Phase | Pt-N  this work | | Pt-N  refs | Pt-O-(ox)  this work | | Pt-O-(ox)  refs | Pt-O (H_2_O, OH)  this work | | Pt-O (H_2_O, OH)  refs |
| --- | --- | --- | --- | --- | --- | --- | --- | --- | --- | --- |
|  |  | Def2^a^ | 6-311++^b^ |  | Def2 | 6-311++ |  | Def2 | 6-311++ |  |
| 1oxpt | Gas phase | 2.093  2.093 | 2.088  2.088 | 2.06 [13 ] | 1,989  1.989 | 1.989  1.989 | 1.99 [ 13] |  |  |  |
|  | Solvent | **2.068**  **2.068** | 2.063  2.064 | 2.06 [12] | **2.035**  **2.035** | 2.034  2.037 | 2.0 [12] |  |  |  |
|  |  |  |  | 2.06 (X-ray) [19] |  |  | 2.01 (X-ray), [19] |  |  |  |
| 2oxpt-h2o | Gas phase | 2.082  2.085 | 2.077  2.082 |  | 1.998  2.002 | 1.998  2.001 |  | 3.599 | 3.550 |  |
|  | solvent | **2.062**  **2.066** | 2.058  2.060 |  | **2.040**  **2.040** | 2.040  2.041 | 2.00, 2.00 [12 ] | **3.705** | 3.715 | 3.46 Pt...OH_2_ [12] |
| 3oxpt-h2o  TS1 | Gas phase | 2.056  2.089 | 2.049  2.085 |  | 1.985  2.469 | 1.985  2.355 | 2.45  [12] | 2.345  Pt-O (H_2_O) | 2.355  Pt-O (H_2_O) | 2.34 Pt..H_2_O  [12] |
|  | Solvent | **2.037**  **2.063** | 2.032  2.057 |  | **2.033**  **2.561** | 2.032  2.567 | 2.03, 2.48  [12] | **2.498**  Pt-O (H_2_O) | 2.505  Pt-O (H_2_O) | 2.44 (Pt…H_2_O)  [12] |
|  | imaginary frequency |  |  |  | **193i cm^-1^** |  | 183i cm^-1^ [12] |  |  |  |
| 4oxpt-h2o | Gas phase | 2.074  2.087 | 2.069  2.083 |  | 2.005  3.251 | 2.004 |  | 2.005  Pt-O (OH) | 2.005, Pt-O (OH) |  |
|  | Solvent | **2.01**  **2.051** | 2.064  2.046 |  | **2.047**  **3.354** | 2.047 | 2.04  12] | **2.043** | 2.042  Pt-O (OH) | 2.08 (Pt-OH_2_)  [12] |
| 5oxpt-2h2o | Gas phase | 2.04 2.03 | 2.01  2.09 |  | 2.047 | 2.046 |  | 2.001 Pt-O (OH)  2.474 Pt-O (H_2_O) | 2.002  Pt-O (OH) |  |
|  | Solvent | **2.054**  **2.046** | 2.052  2.042 |  | **2.051**  **3.405** | 2.049  3.406 | 2.05  [12] | **2.081** Pt-O (H_2_O) | 2.082  4.059 | 2.04 (Pt-OH); 3.48 (Pt…OH_2_)  [12] |
| 6oxpt  TS2 | Gas phase | 2.040  2.083 | 2.079 |  | 2.493 | 2.501 |  | 2.001,  2.047, Pt-O (H_2_O) | 2.001, Pt-O (OH)  2.479, Pt-O (H_2_O) |  |
|  | Solvent | **2.028**  **2.067** | 2.010  2.065 |  | **2.587** | 2.689 | 2.52 Pt-O (H_2_O)  [12] | **2.031** Pt-O (OH)  **2.591** Pt-O (H_2_O) | 2.032, Pt-O (OH)  2.706 (Pt-O (H2O) | 2.03 (Pt-OH), 2.52 (Pt..H_2_O)  [12] |
| 7oxpt-2h2o | Gas phase | 2.087  2.047 | 2.043  2.083 |  | 3.796 |  |  | 1.995, (Pt-O (OH)  2.047, (Pt-O (H2O | 1.996, Pt-O (OH)  2.047, Pt-O (H2O) |  |
|  | Solvent | **2.036**  **2.071** | 2.033  2.068 |  |  | 3.791 |  | **2.032**, (Pt-O (OH)  **2.094**, Pt-O (H2O) | 2.033, Pt-O (OH)  2.093, Pt-O (H2O) | 2.03 (Pt-OH), 2.09 (Pt-OH_2_); [12]  P2 = 7oxpt-2h2o |
| *8oxpt-2h2o | Gas phase | 2.034  2.110 | 2.030  2.107 |  | distant | distant |  | 1.979, Pt-O (OH)  2.098, Pt-O (H2O) | 1.994, (Pt-O (OH)  2.114, (Pt-O (H2O) |  |
|  | Solvent | **2.093**  **2.036** | 2.033  2.089 |  | - | - |  | **2.009** Pt-O (OH)  **2.108** Pt-O (H2O) | 2.012, Pt-O (OH)  2.114, Pt-O (H2O) |  |

*8oxpt-2h2o = [Pt(C_6_H_14_**N**_2_)(**O**H)(H_2_O)]^+^ + HOOC-COO**^-^**

^a^Def2 Basis set (2) see in the text

^b^6-311++ .. Basis set (1), see in the text
